# Genetic Test for the Channelopaties: Useful or Less Than Useful for Patients? (Part II)

**Published:** 2013-05-06

**Authors:** R Romano, V Parisi, F Pastore, A Riccio, L Petraglia, E Allocca, D Leosco

**Affiliations:** 1 Department of Surgery and Cancer, Imperial College London; 2 Department of Clinical Medicine, Cardiovascular and Immunological Sciences, University of Naples “Federico II”; 3 Department of Cardiology, AOU “Maggiore Della Carità”, Novara; 4 Department of Medicine, SUN, Naples

**Keywords:** Channelopaties, Long QT Syndrome, Short QT Syndrome, Catecholaminergic Polymorphic Ventricular Tachycardia, Brugada Syndrome, Genetic Testing

## Abstract

The advanced knowledge about genetic diseases and their mutations has widened the possibility to have a more precise and definitive diagnosis in many patients, but the use of genetic testing is still controversial. Actually, many cardiomyopathies show the availability of genetic testing. The clinical utility of this testing has been widely debated, but it is evident that the use of genetics must be put in a more organic diagnostic pathway that includes the evaluation of risks and benefits for the patient and his relatives, as well as the costs of the procedure. This review aims to clarify the role of genetic in clinics regarding Channelopaties, less frequent but equally important than other Cardiomyopathies because patients can often be asymptomatic until the first fatal manifestation.

## I. INTRODUCTION

The aims of this review are to understand the indication for the execution of genetic tests in patients with Channelopaties and appreciate the difficulties of the interpretation of these tests in the clinical practice. The Channelopaties are less frequent than other cardiomyopathies. The patient is often asymptomatic, even if the first clinical manifestation can be fatal. Among the Channelopaties, the most relevant are the Long QT Syndrome, (LQTS), the Short QT Syndrome (SQTS), the Brugada Syndrome (BrS) and the Catecholaminergic Polymorphic Ventricular Tachycardia (CPVT).

Different Channelopaties with different prognosis could derive from different mutations involving the same gene. Mutations of SCN5a gene can cause the Brugada Syndrome and the LQTS. However, the interpretation of the genetic tests is difficult because they are probabilistic and not deterministic tests. Furthermore, a test could be positive for the presence of a DNA variant with an uncertain clinical significance.

## II. THE CONGENITAL LONG QT SYNDROME

Congenital long QT syndrome (LQTS) is a genetic condition characterized by a prolonged QT interval registered by an ECG and associated with the history of syncope and ventricular tachyarrhythmias, the evidence of T-wave abnormalities, and the high risk of a sudden cardiac death [ 1 ] . The QTc-interval prolongation reflects the prolonged cardiac repolarization. The QT can also be increased by the administration of drugs as antidepressants and antiarrhythmics, or in electrolyte disturbances and structural heart diseases. According to diagnostic criteria proposed by Schwartz et al, the diagnosis is based on clinics [ 2 ] . These criteria ( table 1 ) do not consider the results of genetic testing, even if these tests are extremely crucial to recognize familiar and asymptomatic carriers. Among symptomatic index cases, the untreated 10-year mortality is approximately 50% [ 3 – 5 ] . The QT interval is not the right mean to evidence LQTS because it shortens when the heart rate increases. Thus, it is necessary to use a rate-corrected QT interval to value QT, usually Bazett’s formula is used (QTc= QT/RR 1/2).

There are four clinical types of defined congenital LQTS (cLQTS), including:

- Romano-Ward Syndrome, with autosomal dominant inheritance and prevalence of 1:2,500 (LQTS is present alone) [ 6 ] ;
- Jervell-Lange Nielsen Syndrome, with autosomal recessive inheritance associated with congenital deafness and a rare syndrome;
- Andersen Syndrome, the LQTS is variably presented together with arrhythmias, periodic paralysis, and malformations;
- Timothy Syndrome, an extremely rare disease, characterized by severe LQTS, cardiac and other somatic malformations, and autism.

The acquired syndrome is more frequent than the congenital LQTS, so it is necessary to diagnose LQTS and exclude secondary syndrome.

*The genetic test for LQTS.* Congenital LQTS (Romano-Ward Syndrome) is an autosomal dominant monogenic disorder with variable penetrance. About 85% of the reported cases is inherited parentally while the remaining 15% of patients has a de novo mutation. Actually, mutations have been identified in 13 different genes, although mutations of LQT1, LQT2, and LQT3 are evidenced in the 95% of the cases.

These mutations alter the function of specific ion channels during the normal depolarization, evidenced by a prolongation of the QT interval on the surface ECG.

As shown in table 2 , about 70% of all the LQTS is caused by loss-of-function mutations of the Kv7.1(Iks) potassium channel (LQT1) or the Kv11.1 (IKr) potassium channel (LQT2) while 5%–10% is caused by gain-of-function mutations of the Nav1.5 (INa) sodium channel (LQT3). The LQT8 derived by gain-of-function mutations in the CACNA1C encoding for an L-type calcium channel subunit. The use of the genetic analysis of KCNQ1, KCNH2, and SCN5A must be considered in patients with a LQTS phenotype. However, the analysis of all LQTS genes has not a good evidence and has to be reserved to cases where the clinical suspicion is high, and the previous analysis is negative [ 7 – 8 ] .

Clinical LQTS genetic testing is recommended for any index case when a clinical cardiologist suggests the presence of a LQTS, considering the patient’s history of syncope, the familiar history, the QT interval duration, and the response to catecholamine stress testing. LQTS genetic testing is recommended for asymptomatic patients with QT prolongation (QTc≥500 ms in adults); in patients where the QT prolongation cannot be attributed to secondary conditions associated QT prolongation (e.g. electrolyte, cardiac hypertrophy, bundle branch block, diabetes). LQTS genetic testing may be considered for serial QTc values, on 12-lead ECG≥480 ms in adults, while is not recommended in patients with drug induced LQTS. Furthermore, it must not be performed as part of pre-sports participation or as part of a universal screening protocol. [ 9 ]

A normal QTc on the surface ECG does not exclude the presence of LQTs in relatives and so a genetic-test must be performed in relatives of an index case. After the discovery of a causative mutation, family screening is indicated in all first-degree relatives. Prognostically, the LQT3 mutations are associated with a higher mortality than LQT1 e LQT2 mutations.

Therapeutically, the use of beta blockers is more protective in LQT1 patients, whether in patient with LQT3 it is less protective. The treatment of these patients must consider the use of drugs interacting with sodium current as Flecainide or Mexiletine. The implant of ICD must be performed according the traditional criteria [ 10 – 13 ] .

## III. THE SHORT QT SYNDROME

In 2000 Gussak et al described a new syndrome characterized by a short QT interval shown by a surface ECG, the evidence of malignant ventricular arrhythmias and atrial fibrillation, and the increased risk of sudden death in young people even in the absence of a structural cardiopathy [ 14 ] . This syndrome is rarer than LQTS, whether the real prevalence is unknown. Epidemiological data from Finnish population showed that 97.5% of males had a QTc longer than 348 ms while the value identified for females was 364 ms [ 15 ] . In 2011 Gollob et all defined the clinical criteria for the diagnosis of SQTS. The authors considered 61 patients and made a score using data from these patients ( Table 3 ) [ 16 ] . There is not a widely accepted cut-off for QTc, but generally these patients present a QT interval shorter than 320 ms. The implantation of a defibrillator is the only possible therapy when there is a history of SCD or unexplained syncope. Asymptomatic patients can be treatment with Quinidine, but data are not certain [ 17 ] .

*The genetic test for SQTS.* In contrast to LQTS, the role of genetic testing is limited in SQTS because currently several patients, who have a positive phenotype, have unknown genetic mutations. Three SQTS-susceptibility potassium channel genes showed mutations involved in SQTS: KCNH2, KCNQ1, and KCNJ2. These are usually mutations with a gain-of-function, and repolarization is faster than normally. Obviously, this electrophysiological alteration is not evidenced in all ventricular cells, leading to the creation of a ventricular electrical gradient and so a higher susceptibility to ventricular arrhythmia. This syndrome has an autosomal inheritance, and variable penetrance. After the identification of SQTS-causative mutation in the index case, genetic testing is recommended for first-degree relatives [ 1 ] .

## IV. CATECHOLAMINERGIC POLYMORPHIC VENTRICULAR TACHYCARDIA

Although the diagnosis is usually performed when the patient is about twenty-thirty year old and the first symptom is syncope, one third of patients show symptoms in childhood.

There are two types of CPVT:

- an autosomal dominant type, caused by mutations of the gene encoding for the Ryanodine receptor (RyR2), reported in the 60 % of the patients;
- a rarer type, due to mutations of the gene for cardiac calsequestrin (CASQ2), reported in less than 5% of the patients.

Both show an increased re-uptake of Calcium by sarcoplasmic reticulum with a spontaneous release of Calcium causing delayed after depolarizations. Sometimes the ICD does not protect against Sudden Death because the after depolarizations can determine an significant arrhythmic storm. Drugs such as Beta-blocks and Ca-Antagonist are useful just in a small number of patients [ 19 , 21 ] .

*Genetic-test for CPVT.* More than 100 RyR2 mutations have been discovered for the dominant type of CPVT. They involve specific clusters/regions of the protein, such as FKBP12.6 binding domain, the trans-membrane segments and C-terminus. These regions are extremely large, so some laboratories are not able to evaluate every cluster during a screening test. However, if the first screening is negative, the entire region must be analysed [ 20 ] . CASQ2 mutations are rare. Consequently, only 12 CPVT-associated mutations show to affect this gene. CASQ2 mutations may also cause an autosomal dominant transmission of the phenotype. It is necessary to screen CASQ2 in sporadic RYR2-negative index cases. [ 21 ]

The patients with a non-typical clinical expression rarely have these mutations. So it is unclear if it is useful the screening in patients with IVF and not bi-directional ventricular tachycardia.

Mutations of KCNJ2, encoding for the Kir2.1 potassium channel, and of ANKB, encoding for ankyrin B, can show the presence of clinical symptoms similar to CPVT. However, it is not known the utility of the screen of these genes. The genetic testing for the index case's mutation is indicated in first-degree relatives, even if the phenotype is negative.

## V. BRUGADA SYNDROME

The presence of an abnormal ECG with right bundle branch block pattern and coved-type ST elevation over the right precordial leads characterizes the Brugada syndrome (BS). This disease shows an increased risk of sudden cardiac death in patients with structurally normal hearts [ 22 ] . Patients with BS can be asymptomatic for many years. The first clinical presentation is often a sudden death [ 23 ] . Common symptoms are syncope and agonic breath during the night for the presence of self - terminating ventricular fibrillation episodes [ 23 , 24 ] .

Worldwide, BS is estimated to be responsible for at least 20% of sudden deaths in patients with structurally normal hearts [ 23 , 25 ] . The ECG guides the diagnosis, even if it can show diagnostic features intermittently. Three ECG patterns have been described: Type 1 pattern has a coved ST-segment elevation ≥2 mm, with a negative T-wave; Type 2 has a saddleback ST-segment elevation ≥2 mm, and a positive or biphasic T wave; Type 3 has a coved or saddleback ST-segment elevation type <1 mm. The administration of sodium channel blockers and the placement of right precordial leads over the higher intercostal spaces significantly increases the diagnostic yield unmasking the ECG pattern of the disease [ 26 – 28 ] . Furthermore, ECG pattern can be modulated by a febrile state, autonomic nervous system changes, tricyclic or tetracyclic antidepressants, first-generation antihistamines, alcohol, and electrolytes abnormalities.

The only therapy demonstrated to be successful in BS is ICD implantation. The Consensus on BS [ 23 ] stated that patients with a prior resuscitated sudden death and demonstrated episodes of self-terminating ventricular fibrillation, unexplained syncope, and agonic breath during the night have a Class I evidence for ICD implantation. Unfortunately, the risk stratification lacks in the identification of high risk asymptomatic patients [ 29 – 31 ] .

*Genetic Test for BS.* BS is inherited with an autosomal dominant pattern, but the prevalence is up to 10-fold higher in males, who also exhibit a greater severity of the disease [ 32 ] . The first mutation linked to BS was identified in the SCN5A gene, which encodes for the α-subunit of the sodium channel [ 33 ] . In the last years, several gene mutations have been identified causing loss of-function of the sodium channel [ 34 – 36 ] , producing decreased sodium current, which results in an impaired phase 0 of the action potential and thus producing an arrhythmic substrate. Recently, even mutations of potassium and calcium channels have been related to BS [ 37 ] .

Although many mutations have been identified and the genetic heterogeneity of this syndrome has been established, the precise causal role of the mutation is not fully understood. Indeed, the presence of a mutation is not entirely responsible of the phenotype. Many carriers of mutations present a normal ECG pattern at baseline and after the infusion of a blocker sodium channel [ 38 ] . In addition, a case of identical twins with SCN5A mutations has been reported recently where only one twin displayed the BS phenotype [ 39 ] . These findings suggest that the mutated SCN5A could act as a modifier of others mutated causal genes.

Experts consensus of the Heart Rhythm Society/European Heart Rhythm Association [ 9 ] stated that extensive BS genetic testing can be useful in case of a clinical index of suspicion based on evaluation of the patient’s clinical history, family history, and expressed electrocardiographic phenotype. On the contrary, genetic testing is not recommended in the setting of an isolated type 2 or type 3 BS ECG pattern. Finally, the genetic family screening is recommended after the identification of BS causative mutation in an index case.

## Figures and Tables

**Table 1.** **
Diagnostic criteria for LQTS.
**

** DIAGNOSTIC CRITERIA FOR LQTS **	
ECG findings ^a^	Points

A. QTc ^b^	
- ≥480 ms ^ 1/2 ^	3
- 460–470 ms ^ 1/2 ^	2
- 450 (males)	1
B. Torsades de pointes ^c^	2
C. T-wave alternans	1
D. Notched T-wave in three leads	1
E. Low heart rate for age ^d^	0.5

Clinical history	

A. Syncope ^c^	
- with stress	2
- without stress	1
B. Congenital deafness	0.5

Family history ^e^	

A. Family members with LQTS ^f^	1
B. SD among immediate family members below 30 years	0.5

a In the absence of medications or disorders known to affect these electrocardiographic features.

b QTc calculated by Bazett’s formula, where QTc = QT/ √RR

c Mutually exclusive.

d Resting heart rate below the second percentile for age [Davignon et al., 1980].

e The same family member can not be counted in A and B. Low probability of LQTS is defined by an LQTS score r1 point; an intermediate probability of LQTS is defined by an LQTS score of 2 to 3 points; Z4 points, high probability of LQTS. Modified from Schwartz et al. [1993].

f LQTS, long QT syndrome.

**Table 2.** **
Currently identified LQTS gene mutations
**

** LQTS subtype **	** Gene mutation **	** Protein **	** Note **
LQTS 1	KCNQ1	Alpha-subunit of the slow delayed rectifier potassium channel (Iks)	The first cause of LQTS cardiac events during exercise
LQTS 2	HERG	Alpha-subunit of the rapid delayed rectifier potassium channel (Ikr)	The second cause of LQTS cardiac events occurs during arousal
LQTS 3	SCN5A	Alpha-subunit of sodium channel	Cardiac events during rest/bradycardia Rarely overlaps with Brugada syndrome
LQTS 4	Ankyrin B	Proteins connecting membrane proteins to the spectrin-actin Skeleton	
LQTS 5	KCNE1	MiRP1 beta-subunit of voltage-gated potassium channel	Heterozygote mutation in Romano-Ward syndrome Homozygote mutation results in JLNS
LQTS 6	KCNE2	MinK beta-subunit of the voltage-gated potassium channel	
LQTS 7	KCNJ2	Inward-rectifier potassium channel	Andersen-Tawil syndrome
LQTS 8	CACNA1c	Alpha-1c-subunit of the L-type calcium channel	
LQTS 9	Cav3	Caveolin 3	
LQTS 10	SCN4B	Sodium channel	
LQTS 11	AKAP9	A-kinase anchor protein 9	
LQTS 12	SNTA1	Alpha-1-syntrophin	
LQTS 13	GIRK4	G protein activated inward rectifier potassium channel 4	

**Table 3.** **
Parameters in Symptomatic and Asymptomatic SQTS Cases** . SQTS: short QT syndrome; QTc: Bazett corrected QT interval; QTp: predicted QT interval.

** Parameters **	** Characteristics **	** Symptomatic **	** Asymptomatic **

QT (ms)	n	35	23
	Mean ± SD	270.2 ± 33.3	299.5 ± 34.7
	Median (IQR)	280 (240–297)	300 (285–317.5)
	Overall range	210–334	240–401

QTc (ms)	n	35	26
	Mean ± SD	296.9 ± 22.1	319.8 ± 26.7
	Median (IQR)	302 (288.5–312)	321.5 (304.8–332)
	Overall range	248–381	262–379

QT/QTp (%)	n	35	23
	Mean ± SD	71.9 ± 5.0	77.4 ± 6.6
	Median (IQR)	73 (69.5–75)	78 (74–80)
	Overall range	59–85	64–93
